# Influence of the use of complete denture adhesives on microbial adhesion and biofilm formation by single- and mixed-species

**DOI:** 10.1371/journal.pone.0203951

**Published:** 2018-10-10

**Authors:** Norberto Martins de Oliveira Junior, Danny Omar Mendoza Marin, Andressa Rosa Perin Leite, Ana Carolina Pero, Marlise Inêz Klein, Marco Antonio Compagnoni

**Affiliations:** Department of Dental Materials and Prosthodontics, Araraquara Dental School, Sao Paulo State University (UNESP), Araraquara, Sao Paulo, Brazil; Leibniz-Institut fur Naturstoff-Forschung und Infektionsbiologie eV Hans-Knoll-Institut, GERMANY

## Abstract

**Objectives:**

To verify whether the *Ultra Corega Cream* and *Corega Strip Denture Adhesive* adhesives interfere in the microbial adhesion and biofilm formation by *Candida albicans* and *Lactobacillus casei* in single- and mixed-species settings, and observe whether synergistic or antagonistic relationships between these species occur.

**Methods:**

Specimens made from heat-polymerized acrylic resin (*Lucitone 550*) were fabricated (n = 144) with a circular shape and standardized roughness (3.0 μm ±0.3 Ra) and were divided into three groups: *Without Adhesive* (WA), with *Ultra Corega Cream* adhesive (CA) and *Corega Strips* adhesive (SA). These groups were divided into three subgroups each: *C*. *albicans* single-species, *L*. *casei* single-species and *C*. *albicans* with *L*. *casei* (mixed-species). Microbial adhesion and biofilm formation assays were performed in duplicate at four distinct experimental times (n = 8 per experimental condition). The amount of each microorganism on the surfaces of the specimens was observed by counting of the Colony Forming Units (CFU) per substrate. Additional specimens were characterized by Scanning Electron Microscopy (SEM), with 18 specimens being used in this analysis (n = 18), 2 per experimental condition (n = 2). Two-way ANOVA and Tukey’s test for multiple comparisons were employed, using α≤0.05.

**Results:**

*L*. *casei* (mixed-species) adhered more on the WA substrate than the CA, while *C*. *albicans* (single- and mixed-species) adhered more on the SA. *C*. *albicans*, both single- and mixed-species adhered more than the *L*. *casei* (single- and mixed-species), regardless of the substrate. *L*. *casei* (single-species) formed more biofilm on the WA, but in its mixed cultivation, it had no difference of growth among the tested situations. *C*. *albicans* (single- and mixed-species) formed more biofilm on the SA than the CA, and the fungus formed more biofilm when compared to *L*. *casei*. In general, whenever a species was compared in its single- and mixed-species situation, no statistically significant difference was observed. SEM of biofilm formation assays demonstrated that *L*. *casei* single-species WA formed more biofilm than when the adhesives tested were used, and *C*. *albicans* (both single- and mixed-species) formed more biofilm on the SA than on the CA.

**Conclusions:**

(1) The two denture adhesives tested increased the adhesion of *C*. *albicans* but not of *L*. *casei*; (2) biofilm formation by *C*. *albicans* (single- and mixed-species) was increased on the SA; (3) Relations of synergism or antagonism was not observed between the two microorganisms studied.

## Introduction

Several are the benefits of the use of denture adhesives. These adhesives improve the quality of life of complete denture wearers regarding adaptation, stability, retention, and comfort because these materials can significantly reduce the movement of complete dentures, and provide greater ability to chew and speak [1–5]. The adhesives also act as psychological support to complete denture wearers, promoting greater self-confidence in their use [3, 6–9]. However, many professionals still hesitate to openly recommend denture adhesives because they believe it represents unsuccessful treatment with complete dentures [1,2,6,7].

Denture stomatitis, a fungal infection that affects many complete denture wearers, presents multifactorial etiology and has poor oral hygiene as a major predisposing factor [10–12]. Lack of proper care is pointed out as one of the main contributors to the maintenance of *Candida* spp. in this niche, and, therefore, influence the virulence potential of these fungi [12]. Moreover, the acrylic resin used in complete dentures manufacture presents surface roughness, which often facilitates microbial adhesion and biofilm development when there is precarious hygiene [10]. Among the strategies available to treat denture stomatitis include oral hygiene instruction for complete denture wearers [10–12], the removal of the dentures during sleep, and prescription of antifungals and antiseptics or disinfectants [12]. Currently, the use of probiotics for combating the exacerbated growth of *Candida* species has been speculated in the literature as a coadjuvant of conventional treatment [13]. Probiotics refer to selected strains which are considered as supplements of live microorganisms with a beneficial effect on the host organism.

The association of probiotic species, especially *Lactobacillus*, to *Candida* species has become the target of several current studies that aim for the inhibition of fungal growth [13–21]. For example, the daily consumption of two experimental kinds of cheese supplemented with probiotics (*Lactobacillus acidophilus NCFM* or *Lactobacillus rhamnosus Lr-32*) was effective in inhibiting oral colonization of *Candida* spp. in complete denture wearers [17]. Furthermore, the strains *Lactobacillus rhamnosus HS111* and *Lactobacillus acidophillus HS101* were also probiotic species effective in the treatment of candidiasis of complete denture wearers [15]. Not only in the dental area, but also in gynecology, there is interest in the subject [22–24] since the uncontrolled growth of *Candida* species is also responsible for gynecological infections. However, studies employing the pathogenic *Lactobacillus* species in the treatment of *Candida* infections are scarce [25–27].

A recent study [27] found a correlation between the *Candida* species and the pathogenic *Lactobacillus*; this is evidence that the development of denture stomatitis is more complex than a single fungal infection. According to these authors, both fungi and bacteria play an evident role in the progression of denture stomatitis. Moreover, the *Lactobacillus* species seem to play an important role in the development of denture stomatitis [26], and the pathogenic *Lactobacillus* isolated from the oral cavity can inhibit the growth of *C*. *albicans* through the production of hydrogen peroxide [25].

Further research on the relationship between *Candida* spp. and *Lactobacillus* spp. is necessary to provide greater evidence and clarification on the subject. Moreover, the consequence of the use of denture adhesives with different physical forms of presentation on microbial growth may help to clarify whether these materials influence the interaction between these species. The knowledge of these interactions is essential not only for a better understanding of the pathobiology of microbial infections and interactions but also for the formulation of new strategies and composition of antimicrobial agents that can combat such infections, especially in adhesives used in prosthetic rehabilitation. Therefore, the purpose of the present study was to verify whether the adhesives *Ultra Corega Cream* and *Corega Strip denture adhesives* interfere in the adhesion and biofilm formation by *Candida albicans* and *Lactobacillus casei* in single- and mixed-species settings and to investigate possible synergistic or antagonistic relationships between these species.

## Material and methods

### Preparation of acrylic resin specimens

Specimens (n = 144) were made from metal matrices with ten holes with 15 mm diameter and 3 mm thickness each [28] with Lucitone 550 heat-polymerized acrylic resin (Dentsply International Inc.; USA) for the assays of adhesion and biofilm formation. The surface roughness of the specimens was standardized by the polishing of these with water sandpapers of different granulations (100 and 120, Lixas d’ Água Norton, 100 grade and 120 grade—T 223, Saint-Gobain do Brasil Produtos Industriais e para Construção Ltda.; BRAZIL), and measurement of the surface roughness by a digital profilometer (Mytutoyo Corporation, Model SJ 400; USA), thus seeking to reproduce the average internal surface roughness of denture bases [29]. The profilometer resolution was 0.01 μm, stylus speed was 0.5 mm/second, interval (cutoff length) was 0.8 mm, and transverse length was 2.4 mm [30]. Four measurements were made in each specimen, two on each surface. The average of the readings was determined as the roughness value Ra (μm). The specimens used in the present study had an average surface roughness value (Ra) of approximately 3.0 μm (±0.3) [29]. After standardization of the surface roughness, the specimens were disinfected by exposure to ultraviolet light in a vertical laminar flow chamber (Pachane Indústria e Comércio Ltda.; BRAZIL; Model: PA 115, No 12898), on both surfaces for 20 minutes each and then stored for a maximum of 24 hours in sterile disposable petri dishes (Prolab) which were kept sealed until use.

### Microbiological analyses

The species *C*. *albicans* (strain SC5314) and *L*. *casei* (strain ATCC 4646) were used in the present study. The evaluation of the microbial colonization by the species included adhesion and biofilm formation on the substrates. Substrates consisted in specimens made with acrylic resin Without Adhesive (WA), with adhesive *Ultra Corega Cream* (GlaxoSmithKline Brazil Ltda., BRAZIL; CA) and with adhesive *Corega Strips* (GlaxoSmithKline Brazil Ltda., BRAZIL; SA). Table 1 shows the composition, manufacturer, and batch of the denture adhesives selected for the present study.

**Table 1:** Composition, manufacturer and batch of *Ultra Corega cream* and *Corega strips*.

Adhesive	Composition	Manufacturer	Batch
Ultra Corega Cream	*Sodium/calcium salts of poly (methylvinylether/maleic acid)*, *carboxymethylcellulose*, *mineral oil*, *petroleum jelly*	*STAFFORD-MILLER IRELAND Limited*, *GlaxoSmithKline*, *Youghal Road*, *Dungarvan*, *Co*. *Waterford*, *Ireland*	*R 13462*
Corega Strips	*Sodium carboxymethyl cellulose*, *monocrystalline wax*, *polybutylene*, *polyethylene glycol*	*GlaxoSmithKline Brasil Ltda*. *Estrada dos Bandeirantes*, *8464—Rio de Janeiro—RJ—CNPJ 33*.*247*.*743/0001-10*. *REG*. *ANVISA*: *80141610008*. *Brazilian industry*.	*5S0212V*

### Microbial inocula preparation

The microbial strains used were kept frozen at -80°C and reactivated for use on blood agar plates (*Laborclin*). The plates were incubated at 37°C in 5% CO_2_ for 48 hours. Blood agar plates were used because the colonies from the strains used can be visually differentiated in this culture medium (S1 and S2 Figs). After this period, five colonies of each strain were transferred separately to Tryptone and Yeast Extract (TYE) liquid culture medium supplemented with 1% glucose. These starter cultures were incubated for 16 hours at 37°C and with a 5% CO_2_ environment. The starter cultures were diluted 1:20 and 1:40 in TYE + 1% glucose medium, and incubated until reaching Optical Density (OD) corresponding to the mid-log growth phase which was previously determined (OD_540nm_ = 0.719 ± 0.181 and approximately 8 hours for *L*. *casei*, and OD_540nm_ = 0.97 ± 0.03 and approximately 6 hours for *C*. *albicans*). The OD was verified via a spectrophotometer (CMC Laboratório Ltda.; BRAZIL). The two dilutions were made for each microorganism to allow the selection of one tube per strain, thus ensuring that the dilution with ideal OD was used for the preparation of the *inocula*. The chosen tubes were centrifuged (Centrifugal Eppendorf AG, model 5810R) for 20 minutes (4000 RPM / 4°C). The supernatant was discarded and the same volume of new medium (TYE + 1% glucose) was added, obtaining a microbial concentration of 1x10^6^ CFU/mL for the *inocula* for the adhesion and biofilm formation assays. Inocula of *L*. *casei* only (*L*. *casei* single-species), *C*. *albicans* only (*C*. *albicans* single-species), and *L*. *casei* with *C*. *albicans* were prepared (referred as *L*. *casei* mixed and *C*. *albicans* mixed). *L*. *casei* (single-species) refers to the *L*. *casei* counting in single-species culture, and *C*. *albicans* (single-species) refers to the *C*. *albicans* count in single-species culture. *L*. *casei* (mixed) refers to the *L*. *casei* counting in mixed-species culture, and *C*. *albicans* (mixed) refers to the *C*. *albicans* count in mixed-species culture.

### Adhesives application

The amount of both products *(Ultra Corega cream and Corega Strips)* in each specimen was standardized as approximately 0.050 grams. CA was applied and spread homogeneously directly on the surface of the specimens, forming a thin layer on the surface [7,8,31]. The SA was cut, moistened with sterilized *Milli-q* water and applied on the surface of the specimens [7,8,31]. The process of applying the adhesives to the specimens was done inside the vertical laminar flow chamber, ensuring an aseptic environment. After application, all specimens were again subjected to ultraviolet light exposure for 20 minutes to disinfect the applied adhesives.

### Collection and preparation of saliva

Whole stimulated human saliva was used to incubate the specimens for salivary pellicle formation. The present study was approved by the Institutional Ethical Committee of Araraquara Dental School, Univ Estadual Paulista, UNESP, under registration CAAE: 36069714.7.0000.5416). After an explanation of study procedures, participants needed to explicitly express their desire to participe and sign an informed written consent form.

Stimulated saliva was collected from two volunteers (28 year old male; 38 year old female) who had not eaten for at least two hours before the collection, neither used toothpaste with any active principle other than fluoride or mouthwash with antiseptic substances. Moreover, volunteers did not undergo antimicrobial treatments three months before saliva donation. Saliva was stimulated by chewing a piece of parafilm by the volunteers. A total of 200 mL of saliva was collected in polypropylene tubes. The saliva was then mixed with adsorption buffer (AB buffer– 0.05 M KCl; 0.02 M KPO_4_; 0.02 M CaCl_2_; 0.02 M MgCl_2_) in a proportion of 1:1 (v/v) and 0.1 M PMSF (*phenylmethylsulfonyl fluoride*, *Sigma*‎) was added to a ratio of 1:1000 (0.1 mM PMSF as final concentration). The contents were centrifuged for 10 minutes to 4000 RPM and 4°C. The supernatant was collected (fully clarified saliva) and filtered using a 0.22 μm low protein binding polyethersulfone membrane filter (Rapid Flow, Nalgene). The saliva remained stored at -80ºC until use.

The specimens of all experimental situations (WA, CA and SA) were transferred to 24-well microtiter plates, one disk per well. Next, one mL of sterile saliva was added to each well and the plates were incubated for 30 minutes at 37°C for salivary pellicle formation. After this time, the saliva was removed, and the disks were rinsed with AB buffer.

### Adhesion assays

**Fig 1:**
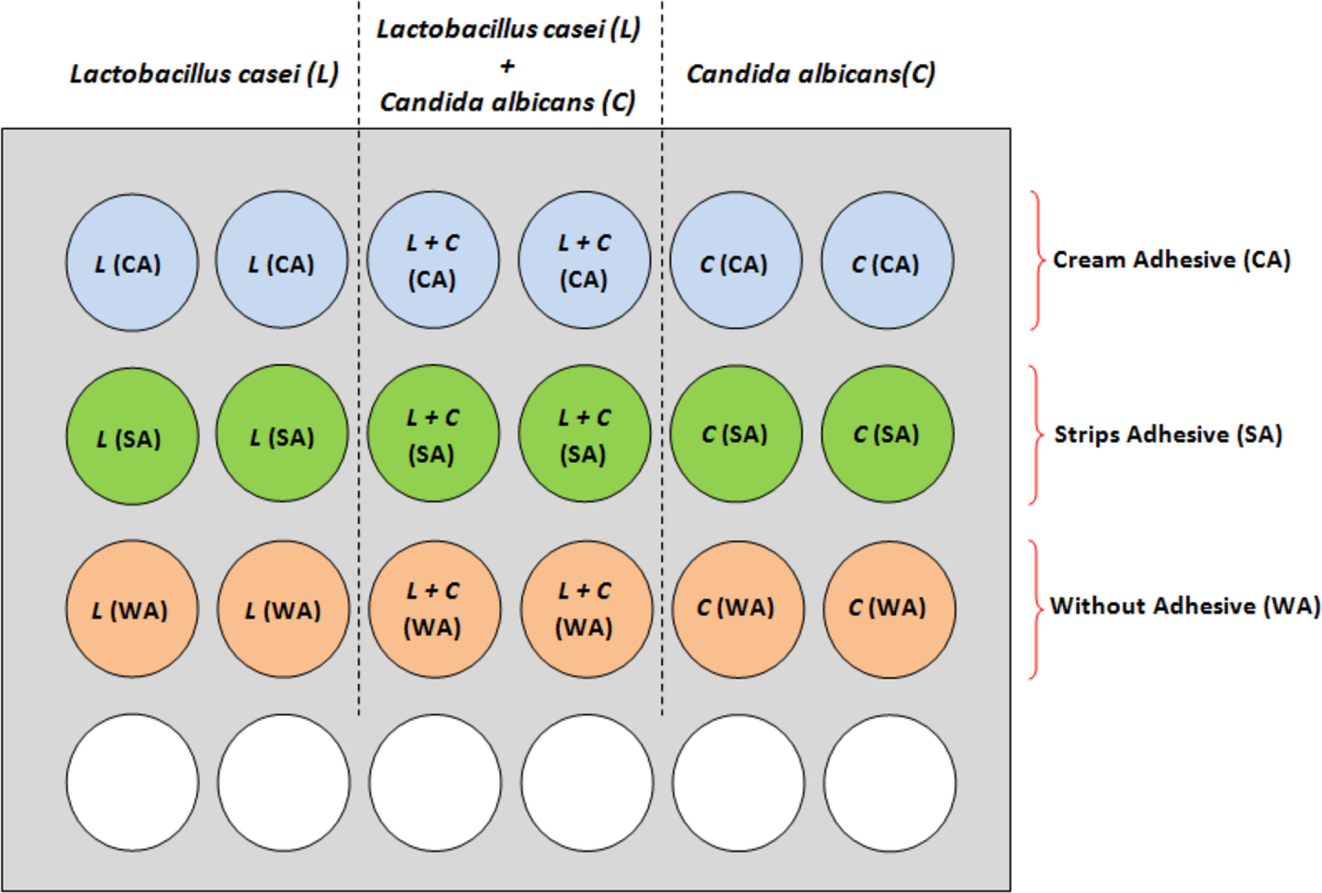
Organization of inocula with different groups of microorganisms and experimental conditions.

A volume of one mL of each *inoculum* (1x10^6^ CFU/mL) was transferred to each well of the microplate containing the distinct specimens, being the inocula with different groups of microorganisms divided as seen in Fig 1.

The microplate was incubated for 90 minutes (37°C and 5% CO_2_) and then the specimens were gently rinsed with saline (0.89% NaCl) to remove any unbound cell. Next, the samples were processed for the quantification of each microorganism adhered to the surfaces by counting the CFU per mL. 90 minutes is the time that characterizes the phase of microbial adhesion. Thus, we considered it was a relevant period for the microbial evaluation since many patients use denture adhesives over this period without replacing them, considering that these products are effective until 3 hours post-insertion [32].

Specifically, each specimen was transferred individually into sterile glass test tubes with two mL of saline (0.89% NaCl) inside with sterile clinical tweezers and in an aseptic environment. The test tubes were sonicated for 11 minutes to remove the adhered cells, followed by 10-fold serial dilution of the microbial suspension per specimen. Serial dilutions of 10^−1^ to 10^−3^ were made. Dilutions were seeded in blood agar plates by the drop technique (10 μL), and these were incubated for 48 hours at 37ºC in a 5% CO_2_ environment and then the number of CFUs were quantified.

### Biofilm formation assays

After the incubation period of 90 minutes (i.e., microbial adhesion phase), to evaluate the biofilm formation, the culture medium (TYE + 1% glucose) was exchanged, and the specimens with the adhered microorganisms remained incubated for an additional 24 hours. Thereafter, the specimens with biofilm were processed in the same manner as described for the adhesion assays, except for the serial dilutions, which were plated up to 10^−4^.

Two replicates were performed per experimental condition in each microplate, considering the different inoculums of microorganisms, the adhesives tested and the proposed assays (adhesion and biofilm formation). Four experiments were carried out on different occasions, totaling an n of 144 and an n of 8 specimens per experimental condition.

### Specimens characterization by Scanning Electron Microscopy (SEM)

SEM was used to observe the adhesion pattern of the cells and the biofilm architecture formed in each experimental condition. After the incubation period, the non-adhered cells were removed by rinsing with saline (0.89% NaCl) and then procedures were performed to fix the adhered cells through immersion of the specimens in 4% paraformaldehyde for 1 hour and subsequently in 70%, 80%, 90% and 99% alcohol solution for 20 minutes each. Washing and immersion procedures were done by moving the specimens from microplate to microplate with distinct solutions, being careful not to remove the cells and to promote the best possible fixation in an aseptic environment. After the fixation process, the specimens were kept in a desiccator for five days for total evaporation of any moisture present in the samples.

During the analysis under the microscope (model JSM-6610LV; Jeol; USA), it was observed that the adhesion samples were hampered by technical problems during the processing, so that the microorganisms could not be found on the surface of the specimens even after repeating the protocol, probably because they were removed from the surface during the wash and rinse. Thus, in the biofilm samples, it was sought to select regions representative of what was generally found on each specimens’ surfaces.

### Statistical analysis

For the CFU count analysis per microorganism (by surface), the obtained data were initially tabulated, and the means, standard deviation, and coefficient of variation were calculated. The data were transformed into log values for better visualization of the results. The normal data distribution was verified, and analysis of variance by two-way ANOVA was employed. Since a significant difference between the groups was indicated, the Tukey’s test for multiple comparisons was used. A significance level of 0.05 was obeyed for all comparisons regardless of the variable (microbial species and substrate). All tests were conducted using the *GraphPad Prism 7* (version 7.03).

## Results

### Microbial population in adhesion and biofilm via CFU quantification

**Fig 2:**
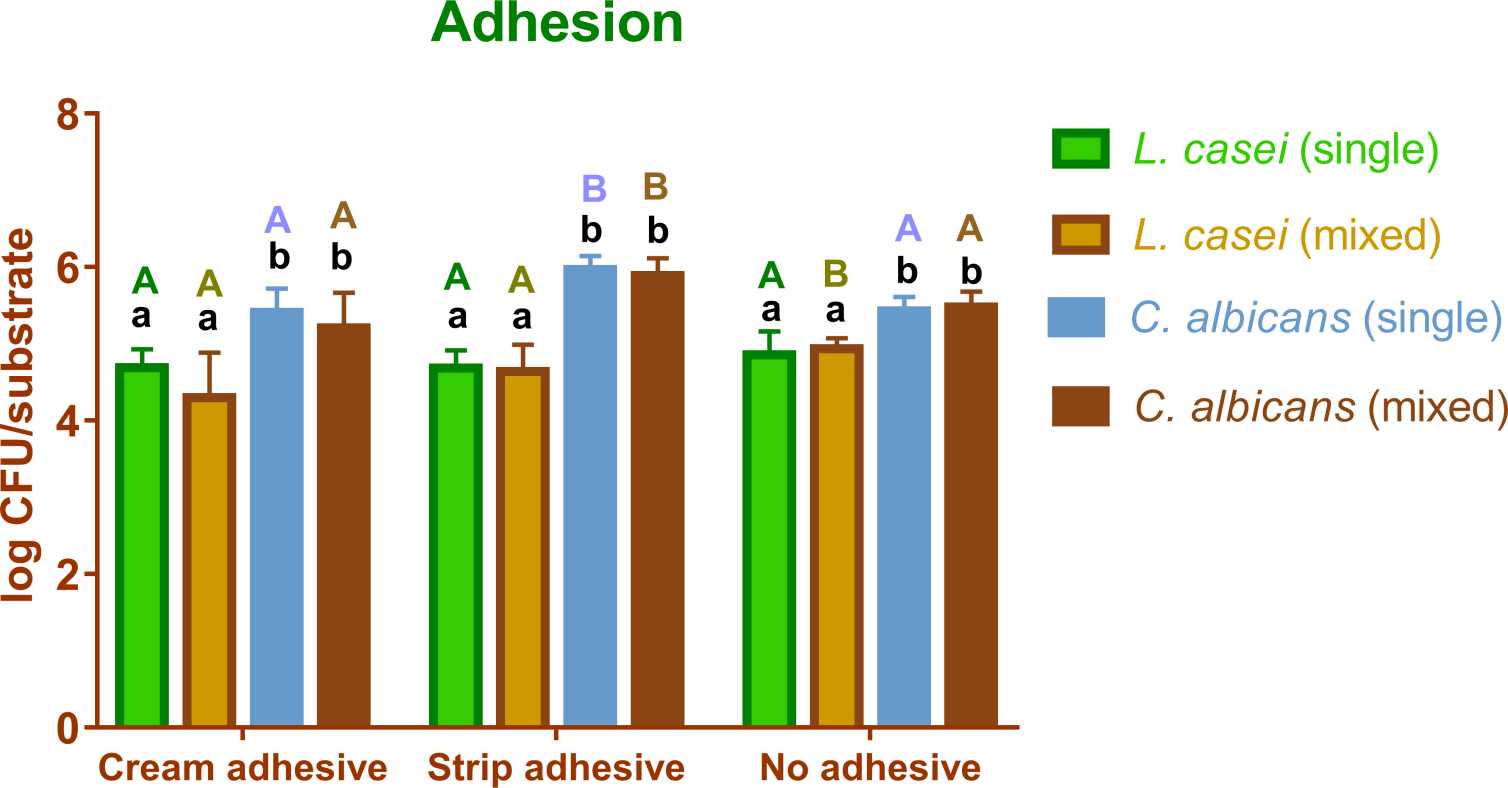
Graph comparing the CFU count during the adhesion experiments in the different experimental conditions. Same low caption letter means no statistical significant difference per surface condition analyzed. Same upper caption letter means no significant difference for species and cultivation condition (single or mixed).

**Fig 3:**
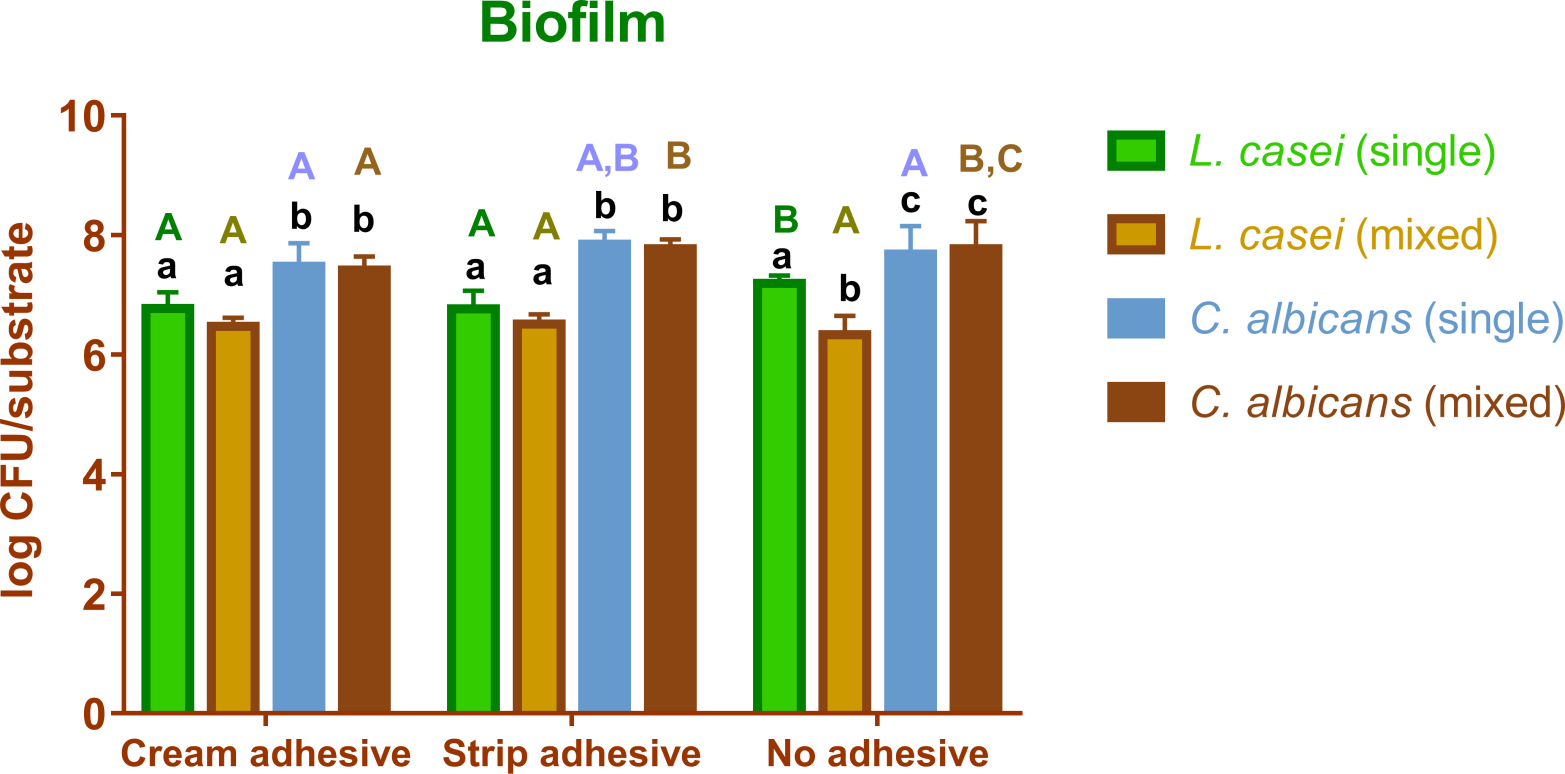
Graph comparing the CFU count during the biofilm experiments in the different experimental conditions. Same low caption letter means no statistical significant difference per surface condition analyzed. Same upper caption letter means no significant difference for species and cultivation condition (single or mixed).

The data concerning the descriptive statistics (mean and standard) for the experimental situations of adhesion and biofilm were tabulated and are represented by Figs 2 and 3. After application of the Tukey’s test for multiple comparisons, a statistically significant difference was detected between several groups according to the experimental situations. The comparisons are demonstrated in Figs 2 and 3.

Regarding adhesion, *L*. *casei*, in general, does not differ its adhesion rate when denture adhesives are used. In a single exception, *L*. *casei* (mixed-species) adhered more on the WA than on the CA, but there were no differences in the amount of adhered cells between the two adhesives tested. *C*. *albicans* (single- and mixed-species) adhered more on SA. Moreover, *C*. *albicans* single- and mixed-species adhered more when compared to *L*. *casei* single- and mixed-species. In all situations (WA, CA and SA), the same species was always compared in its single- and mixed-species situation, and no statistically significant difference was observed.

For biofilm formation, *L*. *casei* (single-species) grew more as a biofilm on WA, and in mixed-species cultivation, no difference in its growth was observed between the distinct surfaces. *C*. *albicans* single-species formed more biofilm on SA than on CA; however, there was no statistically significant difference between SA and WA or between CA and WA. *C*. *albicans* mixed-species presented lower biofilm formation in CA, showing no difference between SA and WA. *C*. *albicans* (single- and mixed-species) formed more biofilm than *L*. *casei* (single- or mixed-species). It was observed that in almost all situations (WA, CA and SA) when the same species was compared in its single- and mixed-species situation, no statistically significant difference was observed. The only exception was *L*. *casei* in the WA cultivation when a higher biofilm formation in the single-species was observed than in the mixed-species.

### SEM characterization

For standardization, images aiming to visualize *C*. *albicans* species were made with 1000 x approximation, as seen in Figs 4–9, and images in which the objective was the visualization of the *L*. *casei* species were done with 2500 x approximation, as seen in Figs 10–15.

**Fig 4:**
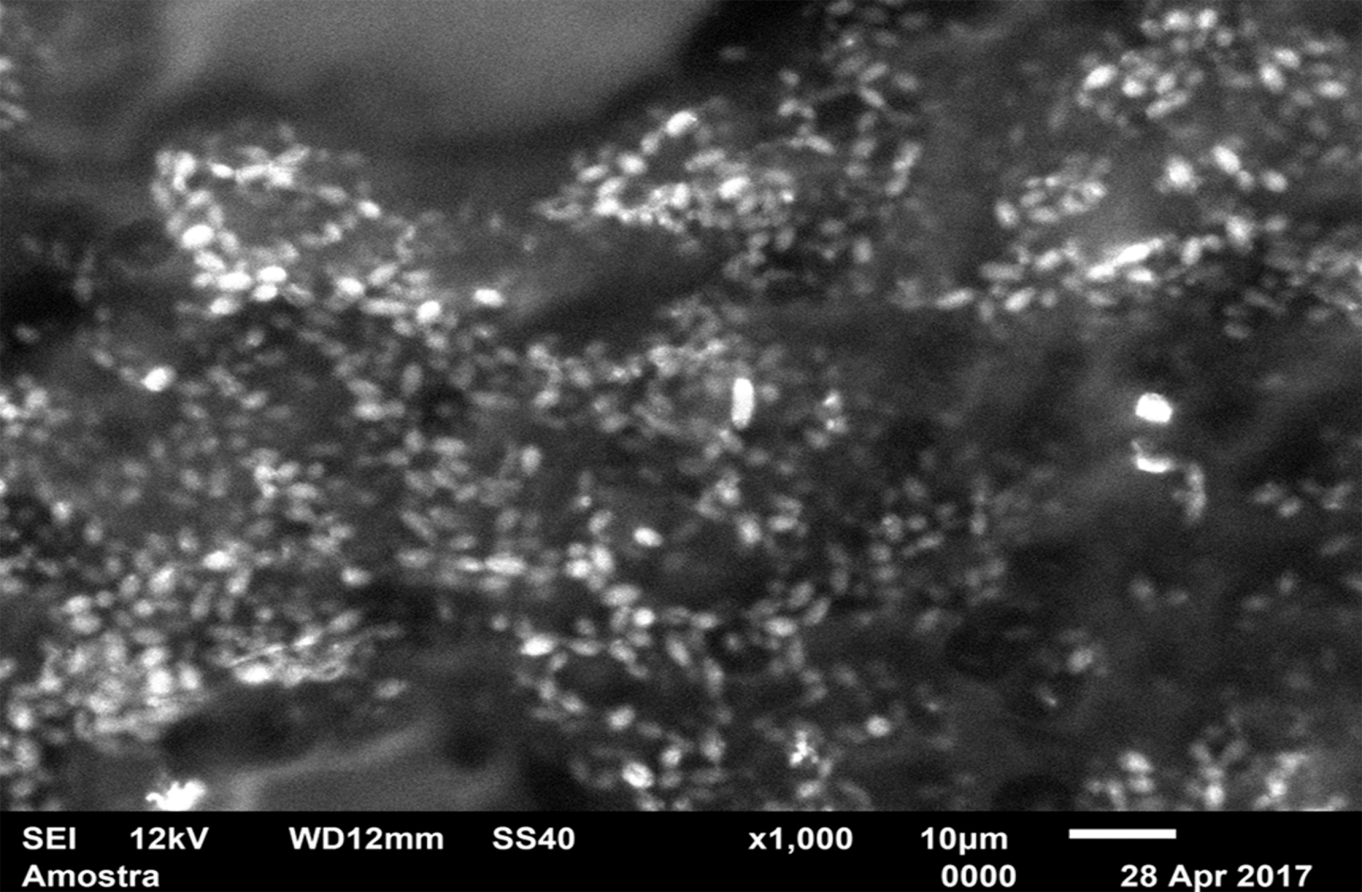
*Candida albicans* single-species with Cream adhesive.

**Fig 5:**
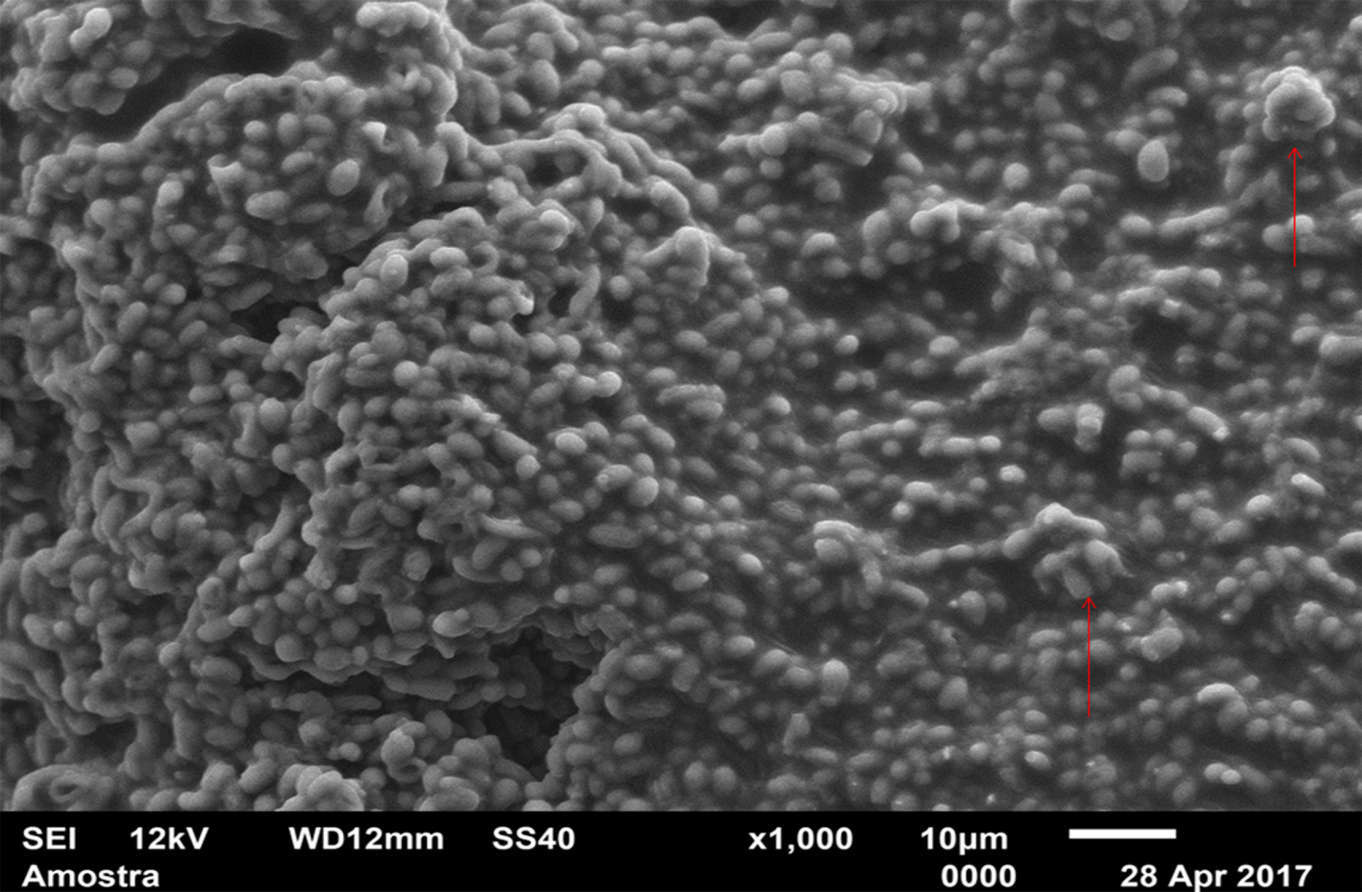
*Candida albicans* single-species with Strips adhesive.

**Fig 6:**
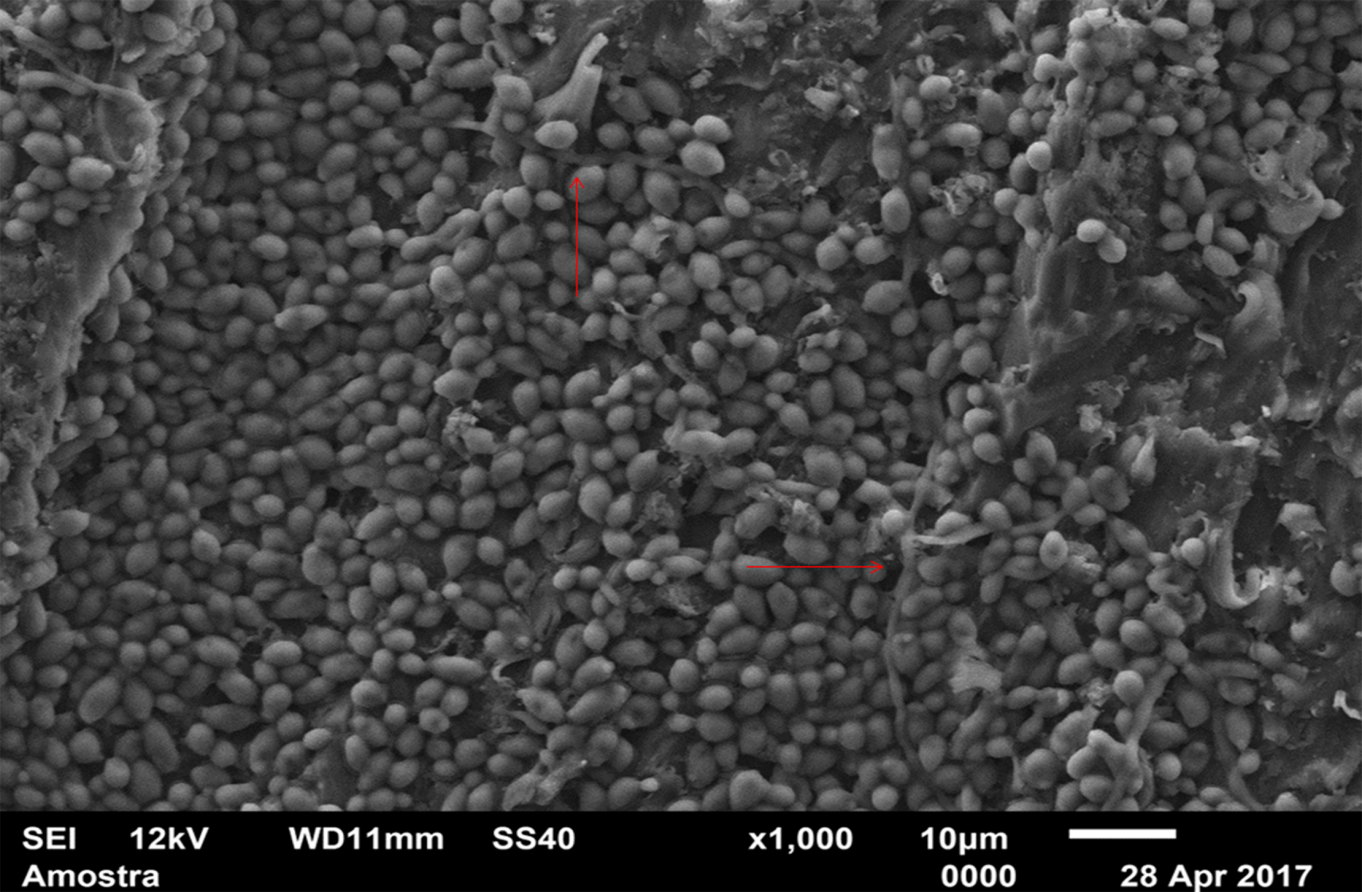
*Candida albicans* single-species Without adhesive.

**Fig 7:**
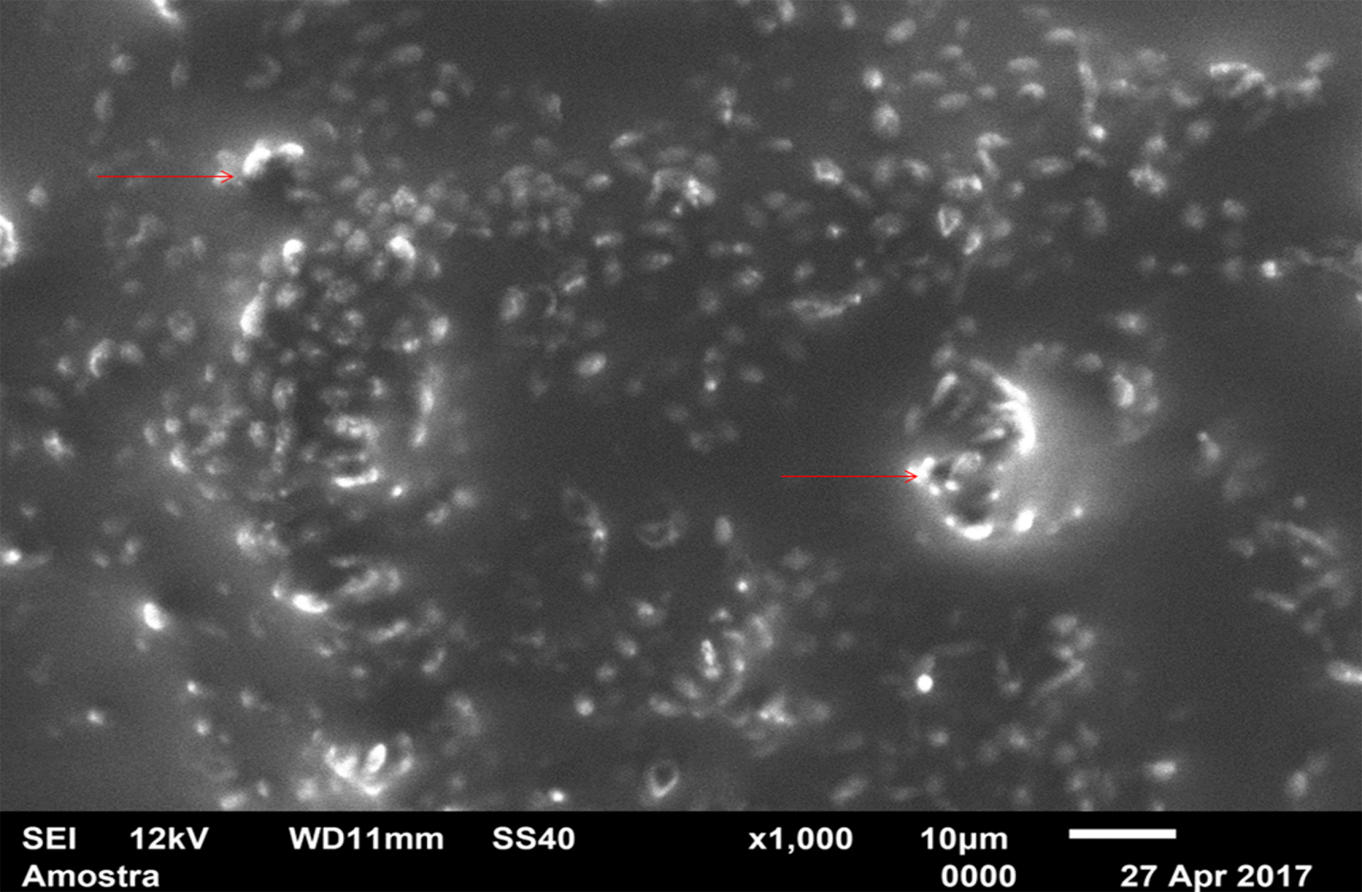
*Candida albicans* mixed-species with Cream adhesive.

**Fig 8:**
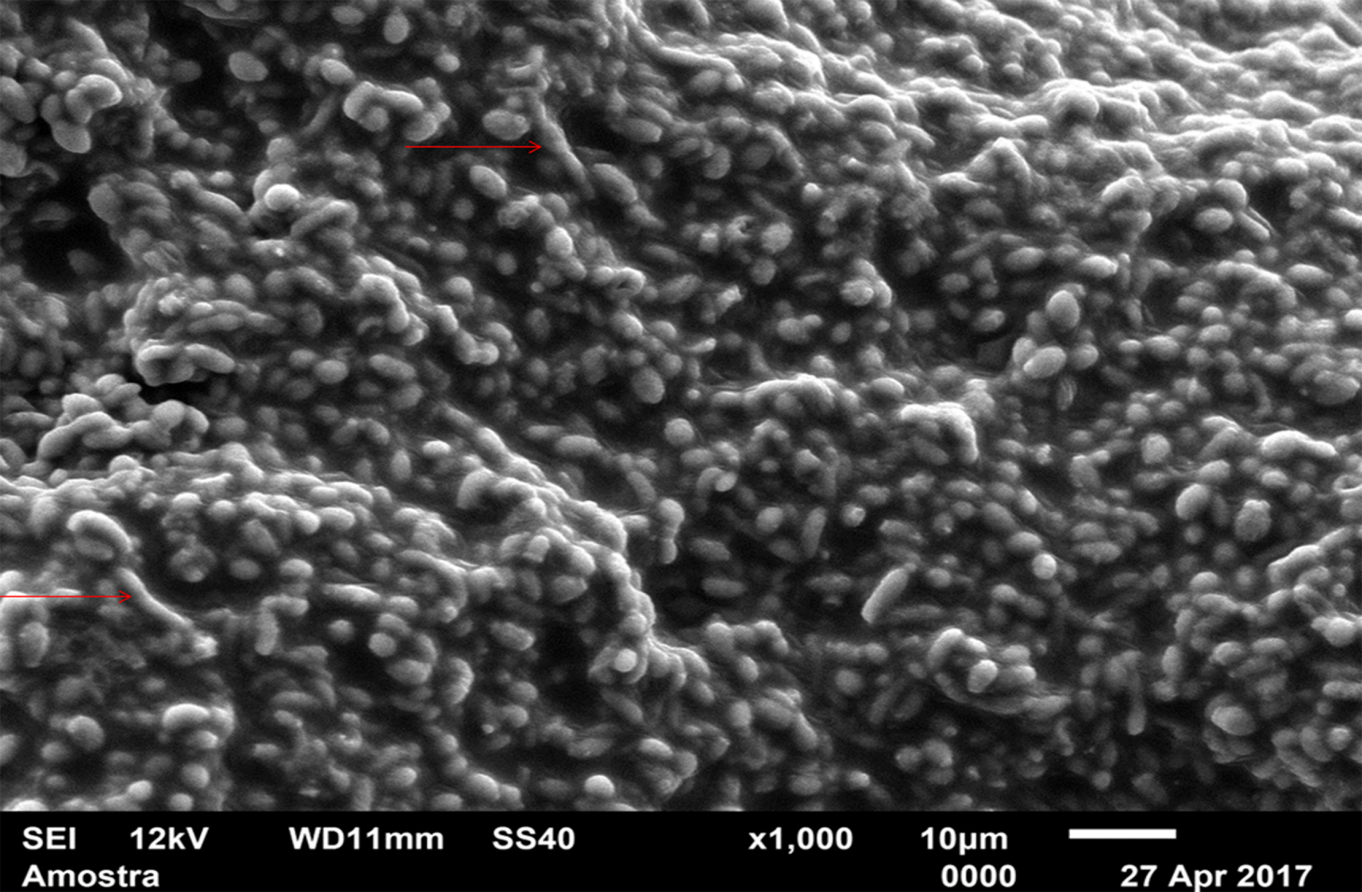
*Candida albicans* mixed-species with Strips adhesive.

**Fig 9:**
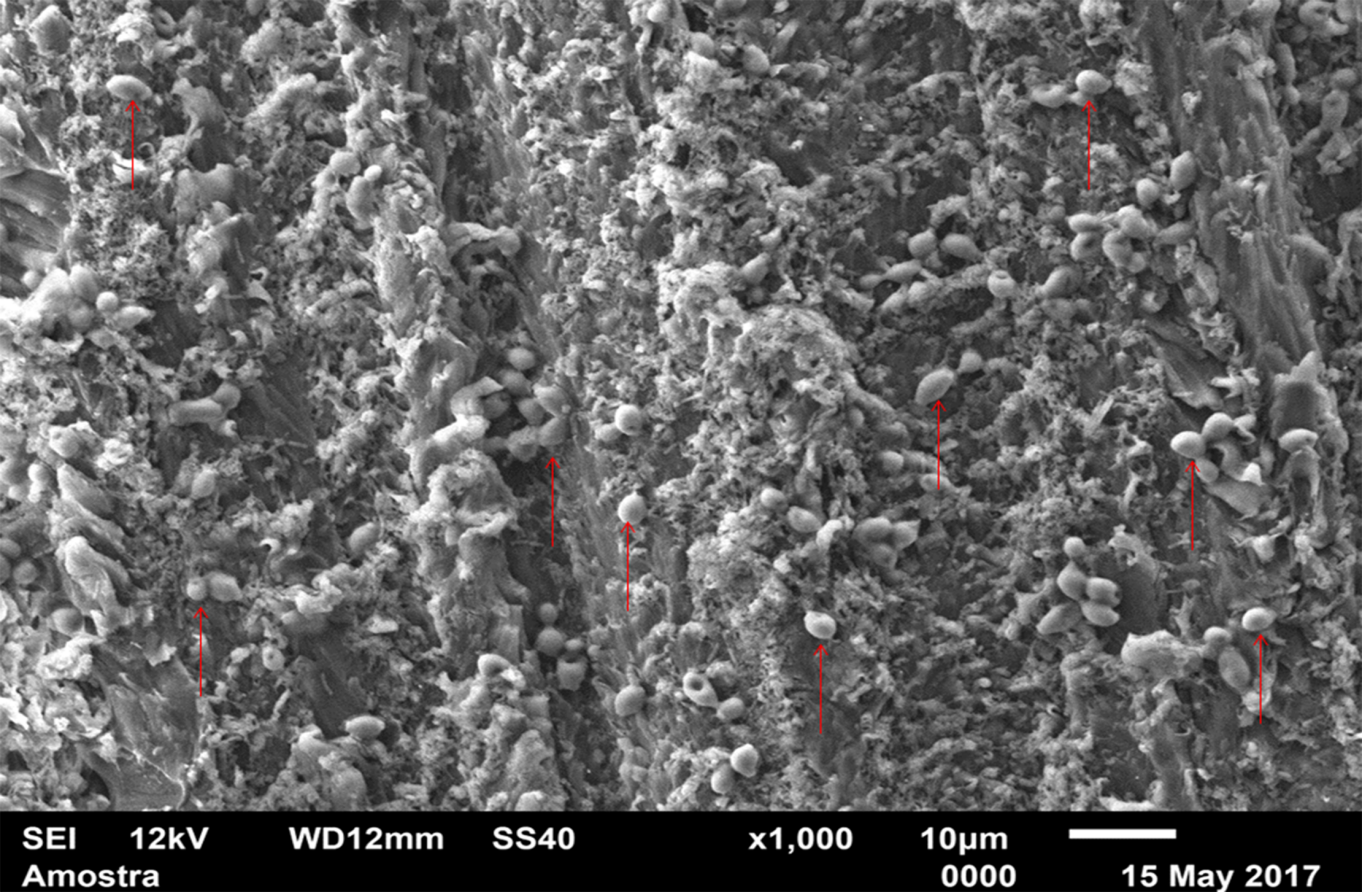
*Candida albicans* mixed-species Without adhesive.

**Fig 10:**
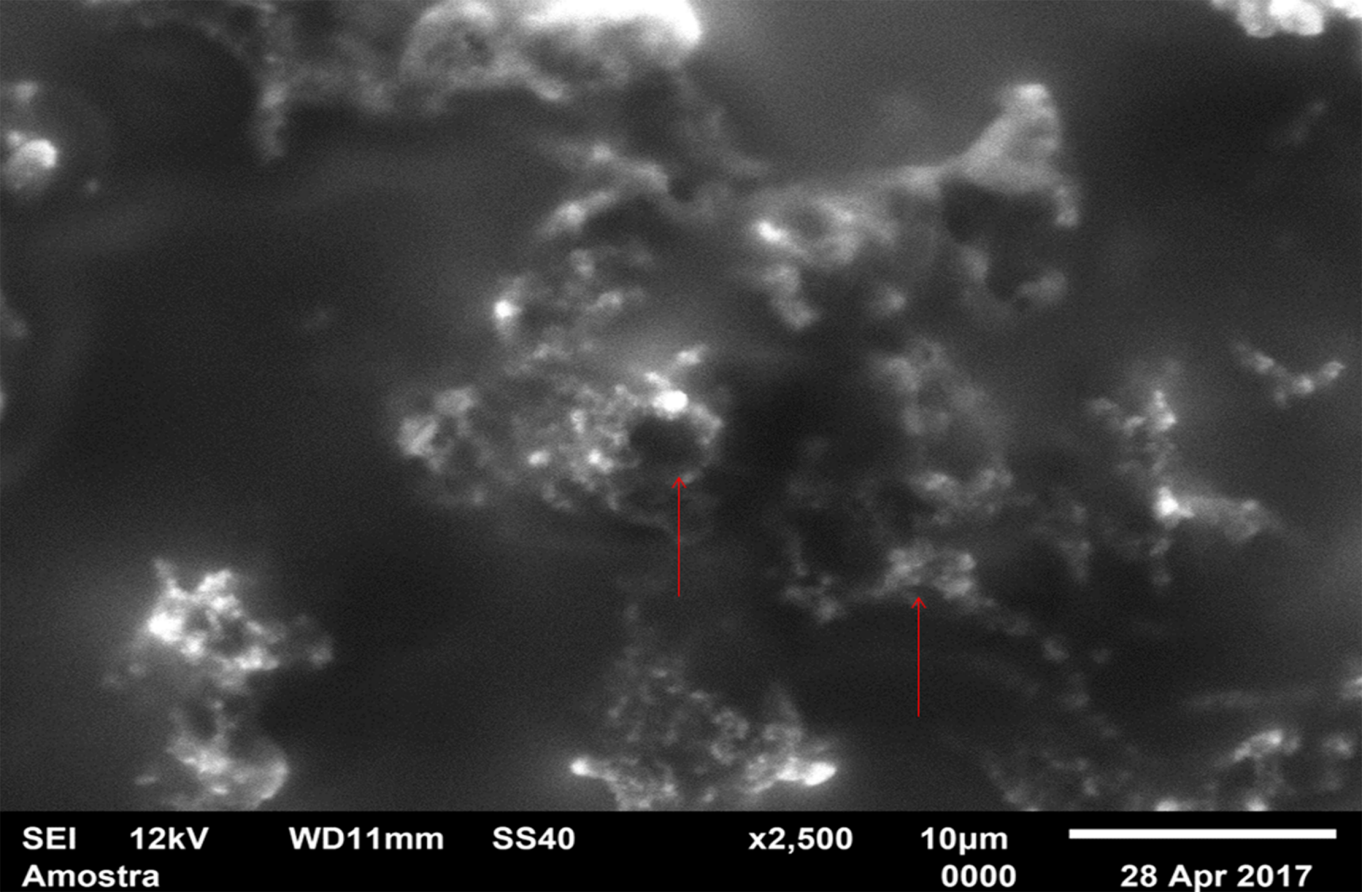
*Lactobacillus casei* single-species with Cream adhesive.

**Fig 11:**
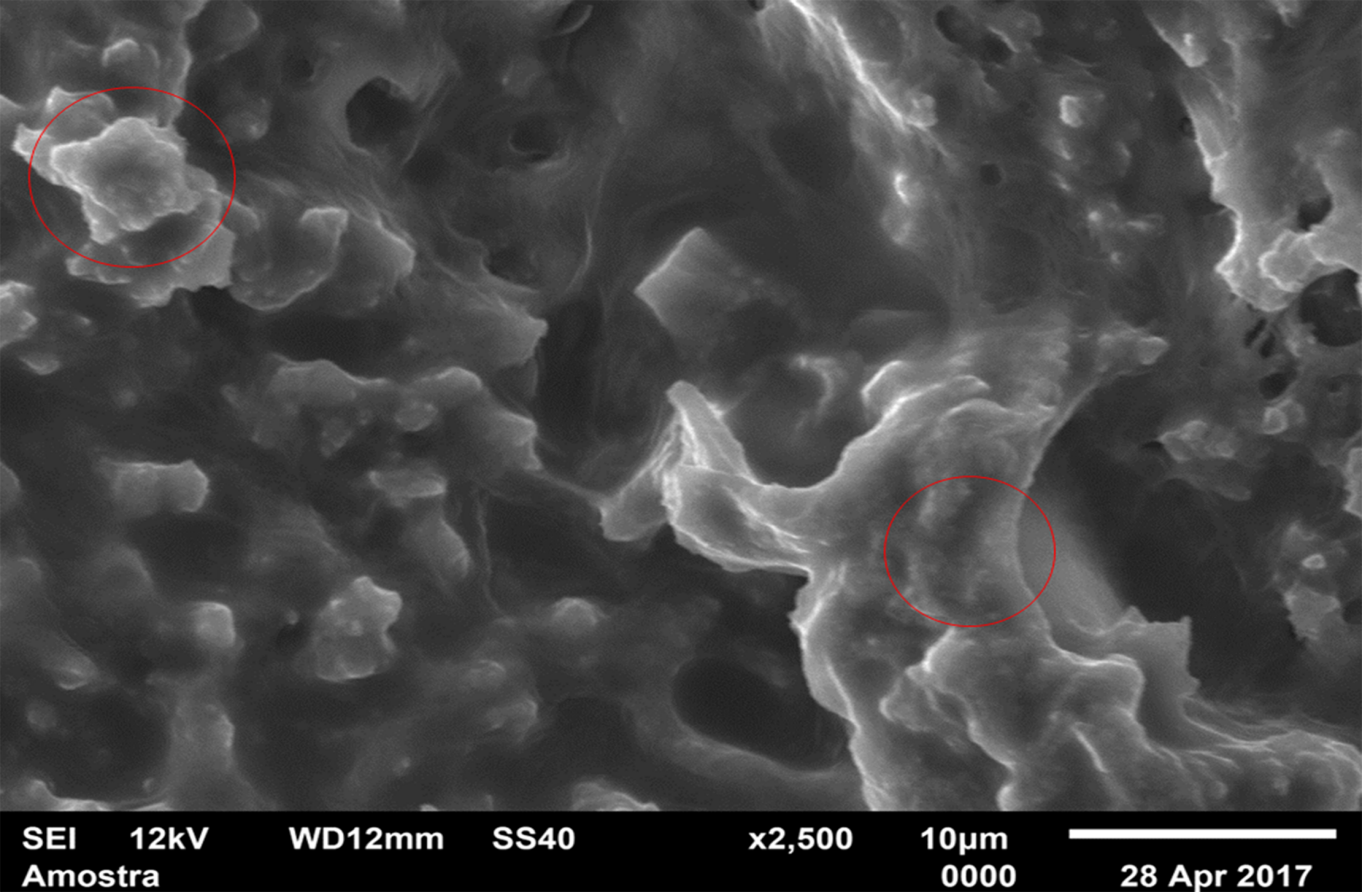
*Lactobacillus casei* single-species with Strips adhesive.

**Fig 12:**
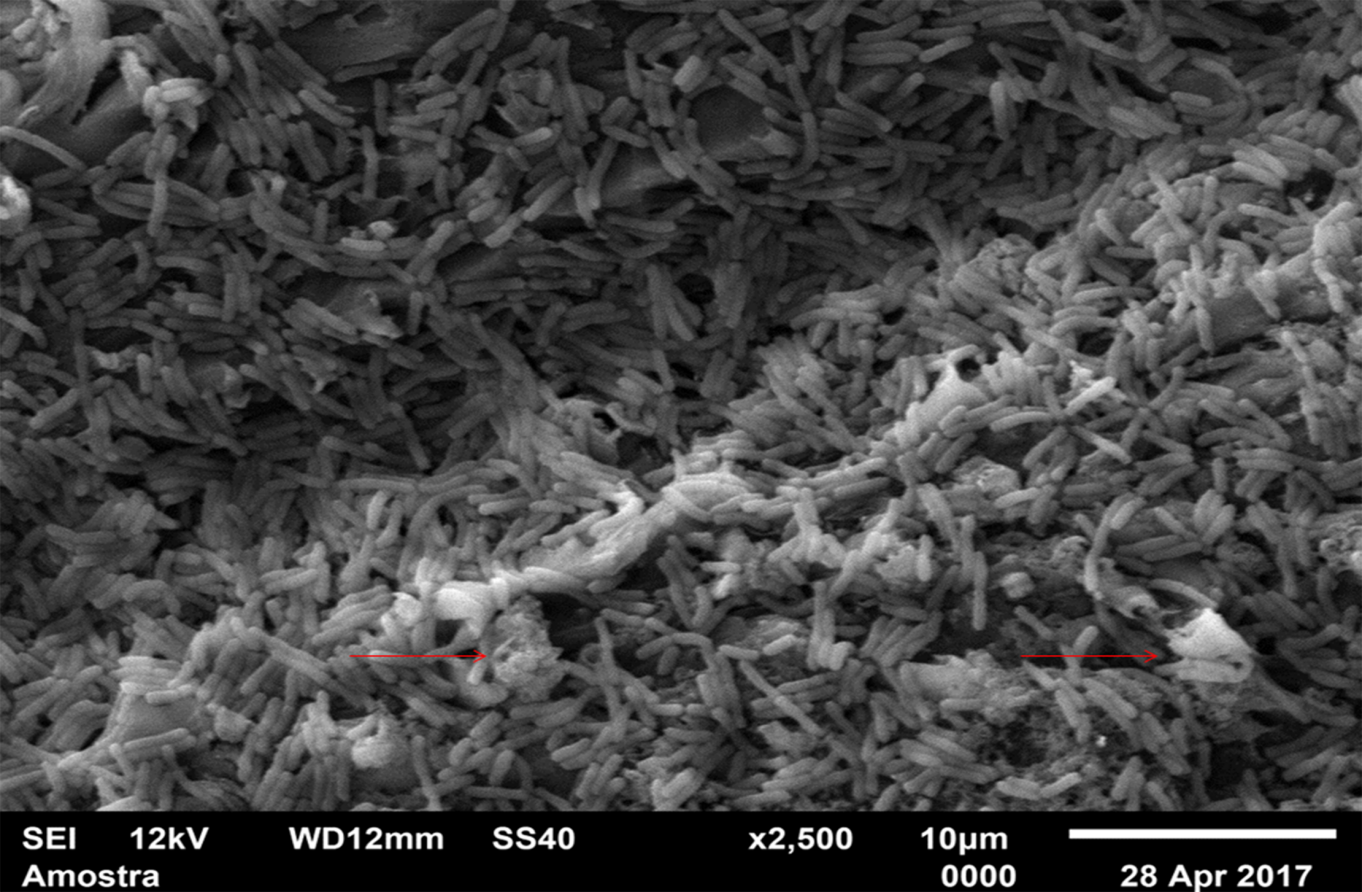
*Lactobacillus casei* single-species Without adhesive.

**Fig 13:**
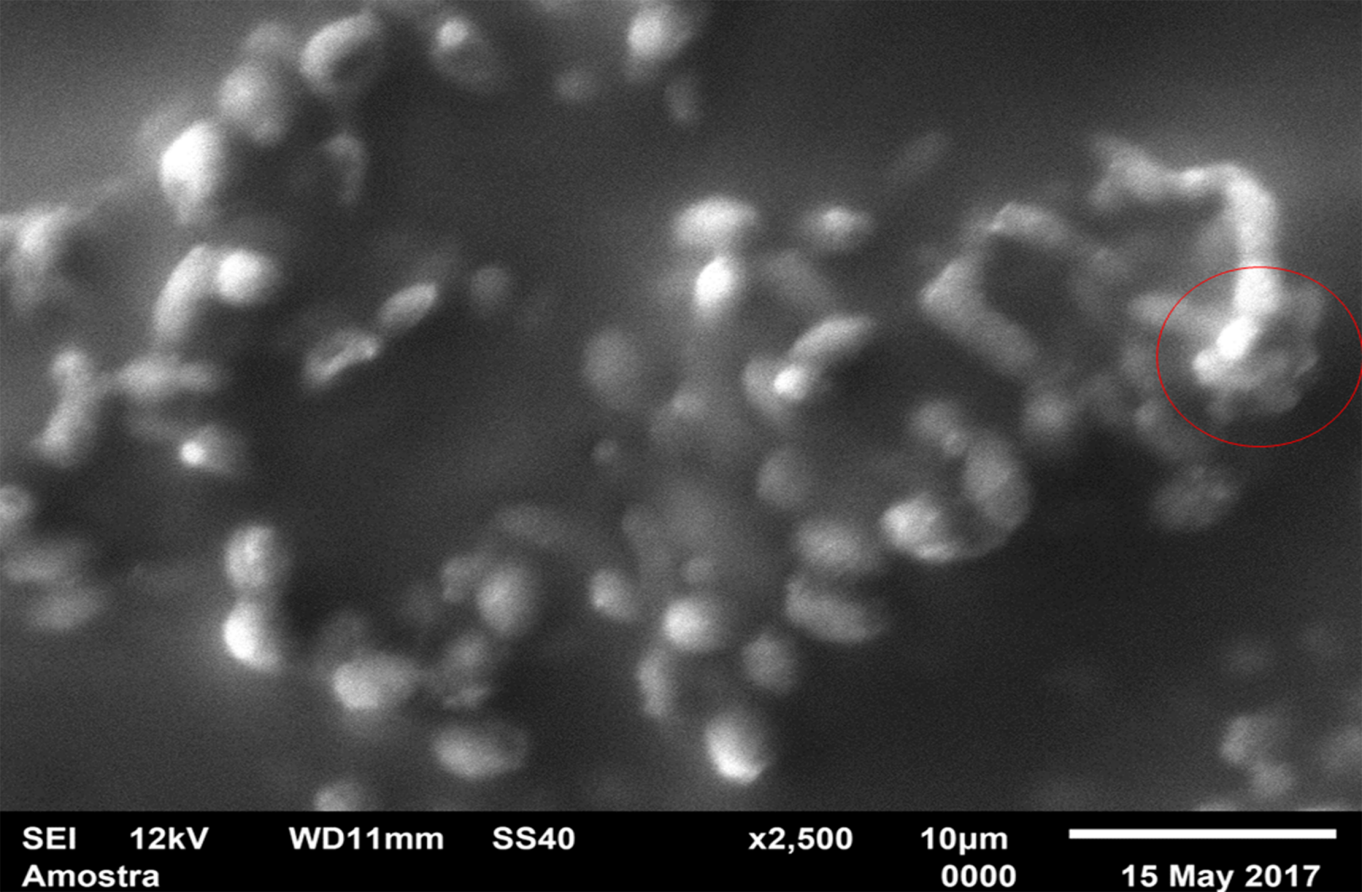
*Lactobacillus casei* mixed-species with Cream adhesive.

**Fig 14:**
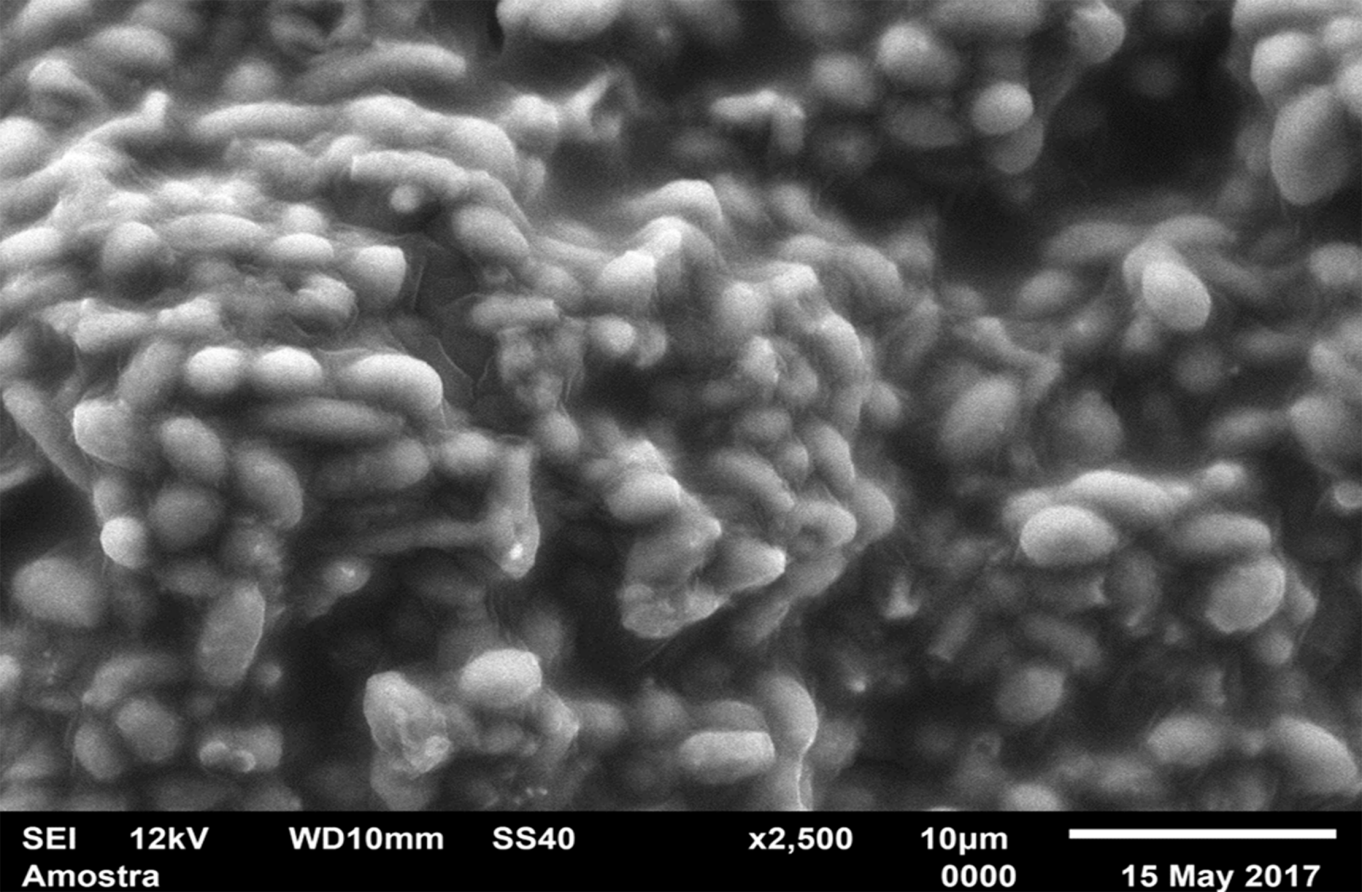
*Lactobacillus casei* mixed-species with Strips adhesive.

**Fig 15:**
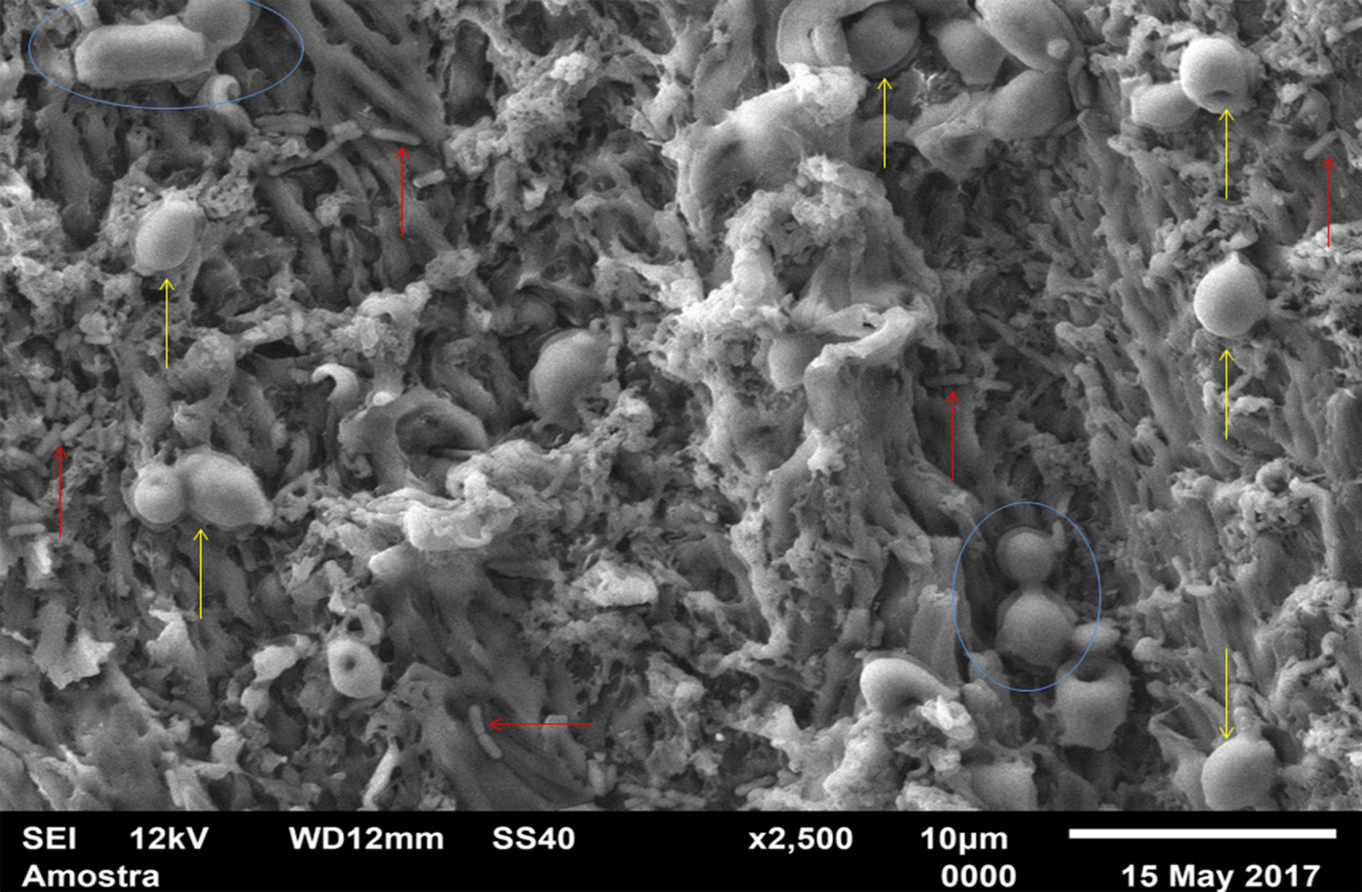
*Lactobacillus casei* mixed-species Without adhesive.

Images of the CA specimens, in general, presented with a "blurred" pattern, are seen in Figs 4, 7, 10 and 13. This observation was probably due to the components of this adhesive, more specifically mineral oil and petroleum jelly, moisturizing and lubricating components, which are not found in the SA composition.

Fig 4 (*C*. *albicans* single-species with CA) shows that *C*. *albicans* biofilms on the CA present cells with yeast morphology only (oval cells), and regions without microbial development. Fig 5 (*C*. *albicans* single-species with SA) shows *C*. *albicans* yeasts wrapped by a "matrix", which is the SA, and a no “blurred” pattern was observed as in the CA images, in Figs 4, 7, 10 and 13. It is possible to note agglomerated microorganisms in some regions (red arrows), characterizing suggestive areas of microcolonies formation, and almost no regions are observed without microbial growth. Most of the observed cells are in the form of yeasts in Fig 6 (*C*. *albicans* single-species WA), but also some extensions (formation of hyphae or pseudohyphae, indicated by red arrows) are observed. Areas with no microbial development are also observed.

The characteristic "blurry" pattern of the samples with the CA is observed in Fig 10 (*L*. *casei* single-species with CA), being difficult to visualize the morphology of the bacterial cells despite the approximation of 2500 x, although it is possible to observe agglomerates (microcolonies indicated by red arrows).

In the *L*. *casei* single-species biofilm formed with the SA, as seen in Fig 11, it is not possible to observe defined cells, but there are areas with a "rough" appearance (indicated by red circles), where it is possible that there are bacteria "covered" by the adhesive (cells localized below the surface). Fig 12 (*L*. *casei* single-species WA) shows bacterial cells organized as a "carpet" on the surface with "peaks" and "valleys" regions which can be visualized because of the surface roughness of the material. There are also areas with an extracellular matrix (visualized as a "net" of polymeric material covering the cells—red arrows).

Fig 7 (*C*. *albicans* mixed-species with the CA) also has a "blurred" appearance. Cells suggestive of yeasts and even microcolonies (red arrows) are observed. But there are areas without microbial development. It is not possible to identify *L*. *casei* in Fig 13 (*L*. *casei* mixed-species with the CA), even with the 2500x increase. The image maintains the characteristic "blurry" pattern of images with the CA. Only yeast-like cells are observed. It is possible to identify a microcolony (red circle). Fig 8 (*C*. *albicans* mixed-species with the SA) shows yeasts enveloped by a "matrix" (SA), and there are some cell extensions in isolated regions (suggestive of hyphal formation indicated by red arrows). A higher number of cells are observed compared to the image made with the CA, which corroborates with the CFU count.

Despite the greater approximation, it is not possible to observe L. casei cells in Fig 14 (L. casei mixed-species with SA) which are "covered" by the adhesive, probably due to the bacterias size.

Despite the greater approximation, it is not possible to observe *L*. *casei* cells in Fig 14 (*L*. *casei* mixed-species with SA) which are "covered" by the adhesive, probably due to the bacterias size. Only yeasts are identified, following the same pattern observed in the 1000 x approximation shown in Fig 8. Fig 9 (*C*. *albicans* mixed-species WA) shows yeasts in isolated regions (red arrows indicate some), and there are also areas without microbial colonization. Fig 15 (*L*. *casei* mixed-species WA) shows *L*. *casei* (red arrows), yeasts (yellow arrows), as well as prolonged cells, characteristic of hyphae (blue circles). There are also areas without microbial colonization. No indicative of a relationship between the microbial species is observed, although the two species can be identified and morphologically differentiated.

## Discussion

Currently, dentistry has advanced techniques and materials that can be used according to each clinical case peculiarities aiming at restoring the loss of dental elements, but the use of conventional complete dentures in oral rehabilitation of completely edentulous patients is still a reality, because of the low cost, systemic limitations or individual choice [33]. Often as an adjuvant of the treatment with conventional complete dentures, denture adhesives may help to increase the retention, and consequently, the overall complete dentures performance by increasing the viscosity of saliva between the complete denture base and the mucosa [6,8,9].

It has been reported that surface characteristics, as surface topography and roughness, are critical factors for adhesion of microrganisms and biofilm formation, mainly *Candida* species [29,34,35]. The use of denture adhesives alters the surface topography of acrylic surfaces [36], which might explain the results of this in vitro study. The two denture adhesives tested increased the adhesion of *C*. *albicans*.

Regarding the type of denture adhesive, the biofilm formation by *C*. *albicans* (single- and mixed-species) was increased on the SA. Denture adhesives can be categorized into soluble (creams, powders and pastes) and insoluble groups (pads and strips). The strips-type denture adhesives (SA) are composed of insoluble polypropylene and cellulose laminae with addition of ethylene oxide and/or sodium alginate, which become viscous when they absorb water from the saliva [37]. This fact could have influenced the biofilm formation of *C*. *albicans* (single- and mixed-species) in this study, which adhered more in the SA (insoluble) than the CA (soluble).

The results of the present study reinforces that the use of denture adhesives inhibited the *L*. *casei* growth, since it has been observed that *L*. *casei*, in general, does not differ its adhesion when adhesives are used. In a single exception, in mixed-species culture, *L*. *casei* adhered more with the WA than with the CA. In addition, the qualitative evaluation (SEM images) corroborates to hypothesize that the use of denture adhesives reduced the microbial growth of the evaluated species.

In both assays of the present study, adhesion and biofilm formation, it was observed that in almost all situations (WA, CA and SA), when the same species was compared in its single- and mixed-species situation, no statistically significant difference was observed, thus reflecting that, in general, no relationship of synergism or antagonism was observed between the two microorganisms. The only exception was *L*. *casei* in biofilm formation WA, where a more significan growth in single- than in mixed-species was observed, which may suggest an antagonistic action of the fungus on the bacterial growth only in this experimental situation.

Furthermore, it was observed that *C*. *albicans*, both as single- and as mixed-species, adhered more in the SA than the WA or CA. Such finding contrasts with a recent study that observed antifungal effects in three physical forms of denture adhesives, and even prolonged effect on the strips adhesive [38]. However, the adhesives trademark used in that study present antimicrobial agents in their composition, which explains the results discrepancy. The inclusion of antimicrobial components in denture adhesives is a relevant recommendation to the manufacturers of these products [39].

*L*. *casei* single-species formed more WA biofilm; however, in their mixed-species cultivation, no difference in growth was observed between the three situations. *C*. *albicans* (single- and mixed-species) formed more biofilm with the SA than with the CA, and there was no difference between the SA and WA. The colonization profile, including the adhesion pattern on a surface or the morphology of biofilms formed on different surfaces, can be analyzed by fluorescence microscopy, SEM or laser confocal microscopy [40]. In the present study, SEM was chosen because the material used in the specimens’ manufacture (acrylic resin) has a high fluorescence index and has the incorporation of fluorophores as a characteristic (due to their porosity), which would hinder the analysis if another option were chosen.

A relevant data from the qualitative evaluation was that, during biofilm formation on the WA, both *L*. *casei* and *C*. *albicans* single-species appeared to have a more intense visual development, which was characterized by the formation of polymeric material by *L*. *casei* and hyphae formation by *C*. *albicans*. The *C*. *albicans* hyphae formation is a characteristic related to tissue invasion, which can cause higher damage to the host and represents an essential step in the candidiasis pathogenesis [41].

Although several studies suggest that different probiotic species of *Lactobacillus* can directly or indirectly act in the fight against *Candida* infections [14,16–20,22–24,42], the present study did not show results suggestive of *C*. *albicans* (strain SC5314) inhibition by the bacterial strain studied (*L*. *casei* ATCC4646), either quantitatively or by SEM analysis. Such results can be explained due to the use, in the present study, of a specific pathogenic bacterial strain with no probiotic capacity demonstrated until then, despite the inhibitory effect of another strain of *L*. *casei* previously reported [20]. In contrast, it was reported by Orsi et al. [42] that certain species of oral *Lactobacillus* (namely *L*. *casei*) demonstrated a stimulatory effect on *C*. *albicans* hyphal growth, impairing the biofilm development.

It is known that there is an association between *Candida* spp. and *Lactobacillus* spp. in the development of denture stomatitis, as demonstrated in clinical samples [27]. Therefore, the choice of strains for *in vivo* studies that aim to confirm probiotic action requires exhaustive execution of *in vitro* studies to determine the best strains with such potential, as well as for such strains not to promote *Candida* spp. virulence but the desired effect of decreasing or inhibiting the pathogenicity of these fungi.

In general, it was also observed that the fungus developed quantitatively superior to the bacterial species, confirming the *C*. *albicans* capacity of adhesion and proliferation in denture materials or hard and soft tissues in the oral cavity [11], as well as the production of complex and heterogeneous biofilm [12,43].

Some of the results found in the quantitative analyses of the present study were reinforced after the characterization of the experimental conditions by SEM. It was observed that the *L*. *casei* single-species WA formed more biofilm than with adhesives, thus reflecting that the adhesives hindered the growth of the bacterial strain when cultured alone. In addition, *C*. *albicans* both single- and mixed-species has formed more biofilm on the SA than on the CA, suggesting that especially in the physical form of strips, the product trademark considers the incorporation of antimicrobials into its composition.

The limitations of this in vitro study were that only one species of Lactobacillus was used, clinical isolates from denture wearers were not tested, and tridimensional imaging of colonization of tested surfaces with denture adhesives, especially CA, was impaired. Moreover, saliva was donated by two healthy non-denture wearers; thus, saliva composition per se could have influenced the colonization, as it may be distinct from partial or total edentulous individuals that use dentures [44,45].

Despite the existence of current studies on the role of denture adhesives on fungal development, especially the *Candida* species [38,39,46–49], further investigations on the interrelationships between bacteria and fungi are still necessary. Thus, a better understanding of the microbiological interactions in the oral cavity of complete denture wearers, especially when using adhesives, may help to consolidate the information found in the present study.

## Conclusions

Within the limitations of the present *in vitro* study, it was possible to conclude that:

(1) The two denture adhesives tested increased the adhesion of *C*. *albicans* but not the *L*. *casei*; (2) biofilm formation by *C*. *albicans* (single- and mixed-species) was increased on the SA; (3) Relations of synergism or antagonism were not observed between the two microorganisms studied.

## Supporting information

S1 Fig *Candida albicans* colony units. (TIF)

S2 Fig *Lactobacillus casei* colony units. (TIF)

## References

[1] Coates AJ. Usage of denture adhesives. J Dent. 2000;28:137–40. 10666972 10.1016/s0300-5712(99)00046-9

[2] Oliveira Junior NM, Rodriguez LS, Mendoza Marin DO, Paleari AG, Pero AC, Compagnoni MA. Masticatory performance of complete denture wearers after using two adhesives: A crossover randomized clinical trial. J Prosthet Dent. 2014,112:1182–7. 10.1016/j.prosdent.2014.05.004 24952882

[3] Panagiotouni E, Pissiotis A, Kapari D, Kaloyannides A. Retentive ability of various denture adhesive materials: an in vitro study. J Prosthet Dent. 1995;73:578–85. 11791272 10.1016/s0022-3913(05)80120-9

[4] Pradíes G, Sanz I, Evans O, Martínez F, Sanz M. Clinical study comparing the efficacy of two denture adhesives in complete denture patients. Int J Prosthodont. 2009;22:361–7. 19639073

[5] Tarib NA, Bakar MT, Murat MDTA, Ahmad M, Kamarudin KH. Masticatory effect and bite force in complete dentures: a study of denture adhesive. Hong Kong Dent J. 2010;7:67–73.

[6] Grasso JE. Denture adhesives: changing attitudes. J Am Dent Assoc. 1996;127:90–6. 8568103 10.14219/jada.archive.1996.0036

[7] Grasso JE. Denture adhesives. Dent Clin North Am. 2004;48:721–33. 10.1016/j.cden.2004.04.002 15261802

[8] Kumar PR, Shajahan PA, Mathew J, Koruthu A, Aravind P, Ahammed MF. Denture Adhesives in Prosthodontics: An Overview. J Int Oral Health. 2015;7:93–5. PMC4516076 26225115

[9] Papadiochou S, Emmanouil I, Papadiochos I. Denture adhesives: A systematic review. J Prosthet Dent 2015;113:391–7. 10.1016/j.prosdent.2014.11.001 25749085

[10] Budtz-Jørgensen E. Ecology of Candida-associated Denture Stomatitis. Microb Ecol Health Dis. 2000;12:170–85.

[11] Gendreau L, Loewy ZG. Epidemiology and etiology of denture stomatitis. J Prosthodont. 2011;20:251–60. 10.1111/j.1532-849X.2011.00698.x 21463383

[12] Salerno C, Pascale M, Contaldo M, Esposito V, Busciolano M, Milillo L, et al Candida-associated denture stomatitis. Med Oral Patol Oral Cir Bucal. 2011;16:e139–43. 20711156 10.4317/medoral.16.e139

[13] Matsubara VH, Wang Y, Bandara HM, Mayer MP, Samaranayake LP. Probiotic lactobacilli inhibit early stages of Candida albicans biofilm development by reducing their growth, cell adhesion, and filamentation. Appl Microbiol Biotechnol. 2016;100:6415–26. 10.1007/s00253-016-7527-3 27087525

[14] do Carmo MS, Noronha FM, Arruda MO, Costa ÊP, Bomfim MR, Monteiro AS, et al *Lactobacillus fermentum* ATCC 23271 Displays *In vitro* Inhibitory Activities against *Candida* spp. Front Microbiol. 2016;7:1722 10.3389/fmicb.2016.01722 27833605 PMC5082230

[15] Ishikawa KH, Mayer MP, Miyazima TY, Matsubara VH, Silva EG, Paula CR, et al A multispecies probiotic reduces oral Candida colonization in denture wearers. J Prosthodont. 2015;24:194–9. 10.1111/jopr.12198 25143068

[16] Jørgensen MR, Kragelund C, Jensen PØ, Keller MK, Twetman S. Probiotic Lactobacillus reuteri has antifungal effects on oral Candida species in vitro. J Oral Microbiol. 2017;9:1274582 10.1080/20002297.2016.1274582 28326154 PMC5328390

[17] Miyazima TY, Ishikawa KH, Mayer M, Saad S, Nakamae A. Cheese supplemented with probiotics reduced the Candida levels in denture wearers-RCT. Oral Dis. 2017; 10.1111/odi.12669 [Epub ahead of print] 28346730

[18] Oliveira VM, Santos SS, Silva CR, Jorge AO, Leão MV. Lactobacillus is able to alter the virulence and the sensitivity profile of Candida albicans. J Appl Microbiol. 2016;121:1737–744. 10.1111/jam.13289 27606962

[19] Rossoni RD, Fuchs BB, de Barros PP, Velloso MD, Jorge AO, Junqueira JC, et al Lactobacillus paracasei modulates the immune system of Galleria mellonella and protects against Candida albicans infection. PLoS One. 2017;12:e0173332 10.1371/journal.pone.0173332 28267809 PMC5340386

[20] Song YG, Lee SH. Inhibitory effects of Lactobacillus rhamnosus and Lactobacillus casei on Candida biofilm of denture surface. Arch Oral Biol. 2017;76:1–6. 10.1016/j.archoralbio.2016.12.014 28063305

[21] Ujaoney S, Chandra J, Faddoul F, Chane M, Wang J, Taifour L, et al In vitro effect of over-the-counter probiotics on the ability of Candida albicans to form biofilm on denture strips. J Dent Hyg. 2014;88:183–9. 24935148

[22] Mezzasalma V, Manfrini E, Ferri E, Boccarusso M, Di Gennaro P, Schiano I, et al Orally administered multispecies probiotic formulations to prevent uro-genital infections: a randomized placebo-controlled pilot study. Arch Gynecol Obstet. 2017;295:163–72. 10.1007/s00404-016-4235-2 27826653

[23] Niu XX, Li T, Zhang X, Wang SX, Liu ZH. Lactobacillus crispatus modulates vaginal epithelial cell innate response to Candida albicans. Chin Med J. 2017;130:273–79. 10.4103/0366-6999.198927 28139509 PMC5308008

[24] Wang S, Wang Q, Yang E, Yan L, Li T, Zhuang H. Antimicrobial compounds produced by vaginal lactobacillus crispatus are able to strongly inhibit candida albicans growth, hyphal formation and regulate virulence-related gene expressions. Front Microbiol. 2017;8:564 10.3389/fmicb.2017.00564 28421058 PMC5378977

[25] Allison DL, Willems HM, Jayatilake JA, Bruno VM, Peters BM, Shirtliff ME. Candida-Bacteria Interactions: Their Impact on Human Disease. Microbiol Spectr. 2016;4 10.1128/microbiolspec 27337476

[26] Bilhan H, Sulun T, Erkose G, Kurt H, Erturan Z, Kutay O, et al The role of Candida albicans hyphae and Lactobacillus in denture-related stomatitis. Clin Oral Investig. 2009;13:363–8. 10.1007/s00784-008-0240-6 19101740

[27] O’Donnell LE, Robertson D, Nile CJ, Cross LJ, Riggio M, Sherriff A, et al The oral microbiome of denture wearers is influenced by levels of natural dentition. PLoS One. 2015;10:e0137717 10.1371/journal.pone.0137717 26368937 PMC4569385

[28] Toda C, Mendoza Marin DO, Rodriguez LS, Paleari AG, Pero AC, Compagnoni MA. Antimicrobial activity of a tissue conditioner combined with a biocide polymer. J Contemp Dent Pract. 2015;16:101–6. 25906799 10.5005/jp-journals-10024-1644

[29] Zissis AJ, Polyzois GL, Yannikakis SA, Harrison A. Roughness of denture materials: a comparative study. Int J Prosthodont. 2000;13:136–40. 11203622

[30] Panariello BH, Izumida FE, Moffa EB, Pavarina AC, Jorge JH, Giampaolo ET. Effects of short-term immersion and brushing with different denture cleansers on the roughness, hardness, and color of two types of acrylic resin. Am J Dent. 2015;28:150–6. 26201226

[31] Felton D, Cooper L, Duqum I, Minsley G, Guckes A, Haug S, et al Evidence-based guidelines for the care and maintenance of complete dentures. A publication of the American College of Prosthodontics. J Am Dent Assoc. 2011;142 Suppl 1:1S-20S. 21282672

[32] Ghani F, Picton DC. Some clinical investigations on retention forces of maxillary complete dentures with the use of denture fixatives. J Oral Rehabil. 1994;21:631–40. 7830198 10.1111/j.1365-2842.1994.tb01178.x

[33] Nicolas E, Veyrune J, Lassauzay C. A six-month assessment of oral health-related quality of life of complete denture wearers using denture adhesive: a pilot study. J Prosthodont. 2010;19:443–8. 10.1111/j.1532-849X.2010.00601.x 20456031

[34] Nikawa H, Jin C, Makihira S, Egusa H, Hamada T, Kumagai H. Biofilm formation of Candida albicans on the surfaces of deteriorated soft denture lining materials caused by denture cleansers in vitro. J Oral Rehabil 2003;30:243–7. 12588495 10.1046/j.1365-2842.2003.01024.x

[35] Yamauchi M, Yamamoto K, Wakabayashi M, Kawano J. In vitro adherence of microorganisms to denture base resin with different surface texture. Dent Mater J 1990;9:19–24. 2098207 10.4012/dmj.9.19

[36] Oliveira MC, Oliveira VM, Vieira AC, Rambob I. In vivo assessment of the effect of an adhesive for complete dentures on colonisation of Candida species. Gerodontology 2010;27:303–7. 10.1111/j.1741-2358.2009.00345.x 19780844

[37] Adisman IK. The use of denture adhesives as an aid to denture treatment. J Prosthet Dent 1989;62:711–5. 10.1016/0022-3913(89)90598-2 2685261

[38] Rajaram A, Manoj SS. Influence of 3 different forms of a commercially available denture adhesive material on the growth of Candida species: An in vitro study. J Prosthet Dent. 2017;118:379–85. 10.1016/j.prosdent.2016.11.015 28222870

[39] Garaicoa JL, Fischer CL, Bates AM, Holloway J, Avila-Ortiz G, Guthmiller JM, et al Promise of Combining Antifungal Agents in Denture Adhesives to Fight Candida Species Infections. J Prosthodont. 2016;doi: 10.1111/jopr.12565. [Epub ahead of print]. 10.1111/jopr.12565 PMC5438910 27870138

[40] Chandra J, Mukherjee PK, Ghannoum MA. In vitro growth and analysis of Candida biofilms. Nat Protoc. 2008;3:1909–24. 10.1038/nprot.2008.192 19180075

[41] Polke M, Hube B, Jacobsen ID. Candida survival strategies. Adv Appl Microbiol. 2015;91:139–235. 10.1016/bs.aambs.2014.12.002 25911234

[42] Orsi CF, Sabia C, Ardizzoni A, Colombari B, Neglia RG, Peppoloni S, et al Inhibitory effects of different lactobacilli on Candida albicans hyphal formation and biofilm development. Journal of biological regulators and homeostatic agents. 2014; 28(4):743–52. 25620183

[43] Zomorodian K, Haghighi NN, Rajaee N, Pakshir K, Tarazooie B, Vojdani, Sedaghat F, Vosoghi M. Assessment of Candida species colonization and denture-related stomatitis in complete denture wearers. Med Mycol. 2011;49:208–11. 10.3109/13693786.2010.507605 20795762

[44] Terrapon B, Mojon P, Mensi N, Cimasoni G. Salivary albumin of edentulous patients. Arch Oral Biol. 1996;41:1183–5. 9134108 10.1016/s0003-9969(96)00095-7

[45] Pajukoski H, Meurman JH, Snellman-Gröhn S, Keinänen S, Sulkava R. Salivary flow and composition in elderly patients referred to an acute care geriatric ward. Oral Surg Oral Med Oral Pathol Oral Radiol Endod. 1997;84:265–71. 9377189 10.1016/s1079-2104(97)90341-3

[46] Kim E, Driscoll CF, Minah GE. The effect of a denture adhesive on the colonization of Candida species in vivo. J Prosthodont. 2003;12:187–91. 10.1016/S1059-941X(03)00050-0 14508740

[47] Leite AR, Mendoza-Marin DO, Paleari AG, Rodriguez LS, Roccia AA, Policastro VB, et al Crossover clinical trial of the influence of the use of adhesive on biofilm formation. J Prosthet Dent. 2014;112:349–56. 10.1016/j.prosdent.2013.11.003 24529654

[48] Oliveira MC, Oliveira VM, Vieira AC, Rambob I. In vivo assessment of the effect of an adhesive for complete dentures on colonisation of Candida species. Gerodontology. 2010;27:303–7. 10.1111/j.1741-2358.2009.00345.x 19780844

[49] Sampaio-Maia B, Figueiral MH, Sousa-Rodrigues P, Fernandes MH, Scully C. The effect of denture adhesives on Candida albicans growth in vitro. Gerodontology. 2012;29:e348–56. 10.1111/j.1741-2358.2011.00478.x 21457296

